# A spontaneous mesenteric hematoma with a fistula between the transverse colon resected by laparoscopic surgery: A case report

**DOI:** 10.1016/j.ijscr.2019.02.007

**Published:** 2019-02-12

**Authors:** Tomoaki Bekki, Takuya Yano, Hiroshi Okuda, Hiroyuki Egi, Shuji Yonehara, Hironobu Amano, Toshio Noriyuki, Masahiro Nakahara

**Affiliations:** a Department of Surgery, Onomichi General Hospital, Onomichi, Hiroshima, Japan; b Department of Gastroenterological and Transplant Surgery, Graduate school of Biomedical and Health Sciences, Hiroshima University, Hiroshima, Japan; c Department of Pathology, Onomichi General Hospital, Onomichi, Hiroshima, Japan

**Keywords:** Spontaneous mesenteric hematoma, Fistula, Hand-assisted laparoscopic surgery (HALS)

## Abstract

**Introduction:**

A spontaneous mesenteric hematoma is rare, and its etiology is unclear. We present a very rare case of spontaneous mesenteric hematoma with a fistula to the transverse colon.

**Presentation of case:**

A 90-year-old male visited our hospital because of lower abdominal pain. The physical examination revealed tenderness in the lower abdomen. The laboratory data revealed anemia and low estimated glomerular filtration rate (eGFR). The abdominal contrast-enhanced computerized tomography (CT) indicated a mass with coexisting low- and high-density areas adjacent to the transverse colon. The mass was diagnosed as a mesenteric hematoma. We selected conservative therapy, because the patient’s condition was stable. After 15 days from discharge, he was admitted to the hospital again for the complaint of bloody stool. A fistula with blood flowing out at the splenic flexure was reported on colonoscopy. In the laparoscopic operative findings, the hematoma adhered to the stomach, pancreas and omentum. A hand-assisted laparoscopic transverse colectomy including hematoma in the mesentery was successful. The pathological reports revealed that a spontaneous mesenteric hematoma formed the fistula to the colon. The patient was discharged without any postoperative complication on day 10.

**Discussion:**

Generally, laparotomy is often chosen for the treatment of mesenteric hematoma. The laparoscopic approach for mesenteric hematoma is useful under the specific condition that the preoperative patient’s condition is stable.

**Conclusions:**

This is the first case of a spontaneous mesenteric hematoma with a fistula to the colon resected safely by laparoscopic surgery.

## Highlights

• A mesenteric hematoma with a fistula to the colon is very rare, and the etiology remains unclear.
• The patients with mesenteric hematoma who are stable can opt for the non-operative treatment.
• Laparoscopic surgery may be a useful treatment option compared to open surgery with respect to postoperative recovery.

## 1 Introduction

The most common cause of a mesenteric hematoma is trauma [1]; whereas, mesenteric vascular diseases, such as an aneurysm and vasculitis [2,3] generally result from non-traumatic causes. A spontaneous mesenteric hematoma is diagnosed when none of the clinical or pathological findings is confirmed.

A mesenteric hematoma is identified on imaging modalities, such as abdominal contrast-enhanced CT. An angiography is also useful for the diagnosis of the origin of the bleeding point. A spontaneous mesenteric hematoma can be treated conservatively, if the hemorrhage can be controlled [4].

A spontaneous mesenteric hematoma is a rare condition, and its detailed etiology is unknown [4]. In this report, we present a case of a spontaneous mesenteric hematoma with a fistula to the colon resected by a laparoscopic operation. We report this case with a review of the literature. This work has been reported in line with the SCARE criteria [5].

## 2 Case presentation

**Fig. 1:**
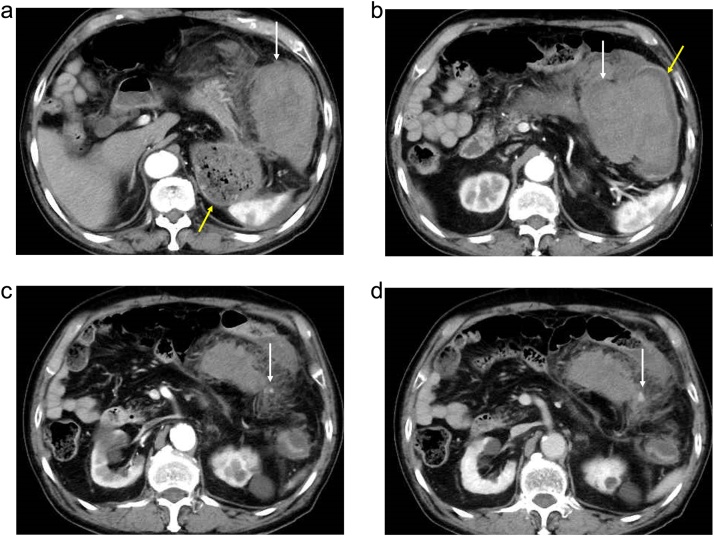
Abdominal contrast-enhanced computed tomography (CT) findings. (a) A mass (white arrow) with coexisting low- and high-density areas with a maximum diameter of 120 mm adjacent to the stomach (yellow arrow). (b) A mass (white arrow) adjacent to the transverse colon (yellow arrow). (c) Extravasation (white arrow) in the mass was observed in the arterial phase. (d) Extravasation (white arrow) spread in the portal phase.

**Fig. 2:**
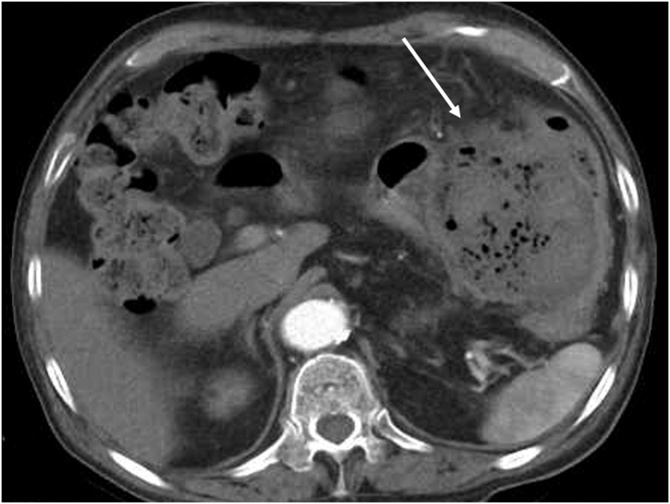
Abdominal contrast-enhanced computed tomography (CT) findings. An air-filled mass (white arrow) measuring 90 mm in diameter.

**Fig. 3:**
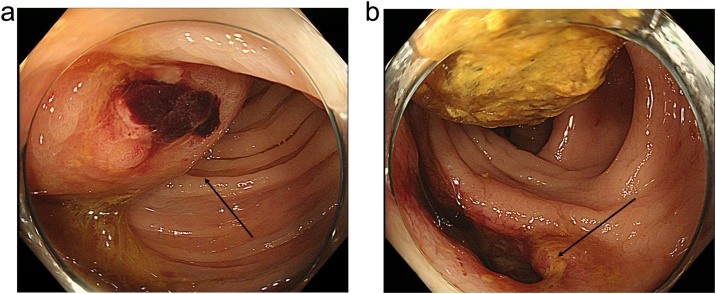
Colonoscopic findings. (a) Many ulcers (black arrow) were confirmed at the transverse colon at the splenic flexure. (b) A fistula (black arrow) with the blood flowing out at the transverse colon at the splenic flexure.

**Fig. 4:**
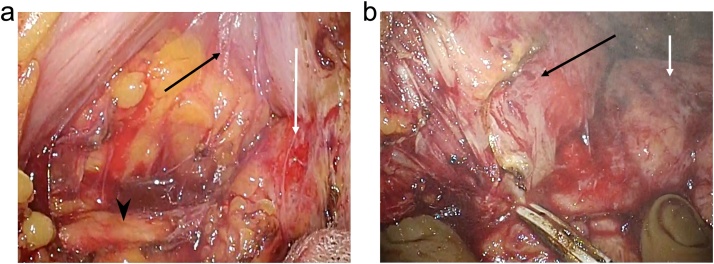
Intraoperative findings. (a) The mass (white arrow) was confirmed in the mesentery of the transverse colon at the splenic flexure; adhesion developed between the stomach (black arrow), pancreas (black arrowhead), and omentum because of high inflammation. (b) Released adhesion of the stomach (black arrow) and hematoma (white arrow) while touching the hematoma.

**Fig. 5:**
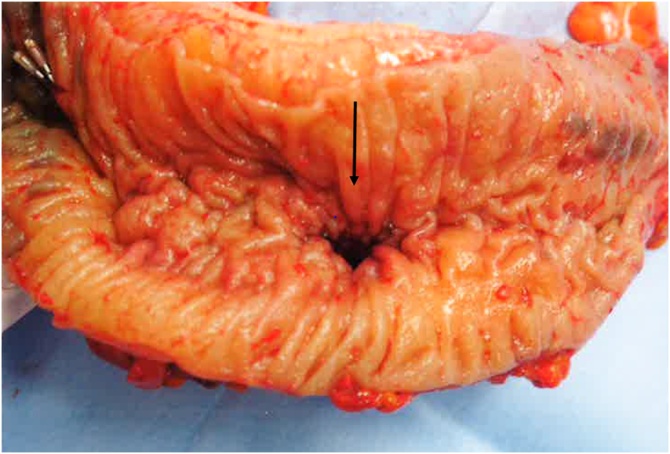
Macroscopic pathological findings. The fistula (black arrow) was found between the transverse colon and the mesentery.

**Fig. 6:**
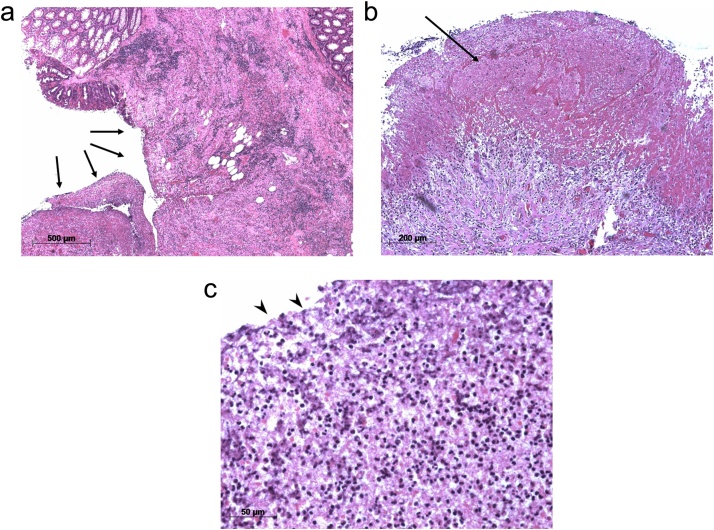
Histopathological findings. (a) The aperture of the fistula. There were no epithelial cells in the fistula (black arrow). (Hematoxylin-eosin stain, original magnification ×50). (b, c) Hematoma component (black arrow) and numerous lymphocytes (black arrowhead) were found in the mesenteric cavity. There were no findings of mesenteric aneurysm and arterial sclerotic change. We diagnosed that a hematoma and an abscess coexisted within the mesenteric cavity. (b. Hematoxylin-eosin stain, original magnification ×100; c. Hematoxylin-eosin stain, original magnification ×400).

A 90-year-old male was admitted to our hospital for the complaint of lower abdominal pain. The physical examination revealed tenderness in the lower abdomen; however, he had no symptom of peritoneal irritation. He presented with comorbidities of hypertension, hyperlipidemia, and hyperuricemia. He had no history of surgery and trauma. The laboratory data revealed anemia and low estimated glomerular filtration rate (eGFR) (hemoglobin level: 10.3 g/dL; eGFR: 35 mL/min/1.73m^2^). All the other data were within the normal range. The abdominal contrast-enhance CT indicated a mass with coexisting low- and high-density areas with a maximum diameter of 120 mm adjacent to the stomach and transverse colon (Fig. 1a,b). An extravasation was observed in the mass in the arterial phase, and it spread in the portal phase (Fig. 1c,d). The mass was diagnosed as a mesenteric hematoma. We selected conservative therapy, because the vital signs were stable and the anemia was mild. In addition, there was an improvement in the abdominal pain. On the 2nd day of the admission, the anemia progressed (hemoglobin volume: 9.5 g/dL); therefore, the abdominal contrast-enhance CT was performed again to confirm the findings. It was observed that the density of the mass had decreased; the size of the mass had reduced; and the extravasation was not present. The patient’s hospitalization course was uneventful. He was discharged on the third day after admission due to the improvement of anemia (hemoglobin volume: 11.4 g/dL) and his strong hope. After 7days from discharge, we checked his laboratory data in the outpatients clinic; showed no anemia progress (hemoglobin level: 11.4 g/dL). After 15 days from discharge, he was admitted to the hospital again for the complaint of bloody stool. He had no other complaints, such as fever and abdominal pain. The laboratory data revealed that his white blood cell (WBC) count was within the normal range; but, the C-reactive protein (CRP) level was elevated (18.36 mg/dL); the hemoglobin level was 10.5 g/dL, which was not different from that during the first visit. On the abdominal contrast-enhanced CT, an air-filled mass measuring 90 mm in diameter was observed (Fig. 2). We diagnosed it as a mesenteric hematoma with a fistula between the transverse colon. We thought that the etiology, malignancy or not, change operation methods. We decided to perform a colonoscopy on the following day. The colonoscopic findings revealed many ulcers and fistulae with the blood flowing out at the transverse colon at the splenic flexure (Fig. 3a,b). There was no diverticulum. We selected an antibacterial drug therapy before the operation, because he had no other complaints and his vital signs were within the normal range. Subsequently, the patient underwent a laparoscopic transverse colectomy. The mass was confirmed in the mesentery of the transverse colon at the splenic flexure, and it had formed adhesions between the stomach, pancreas, and omentum. We changed the procedure to hand-assisted laparoscopic surgery (HALS) to perform the operation safely (Fig. 4a,b). The total operation time was 248 min, and the total intraoperative blood loss was 268 ml. Macroscopically, the fistula was found between the transverse colon and mesenteric hematoma (Fig. 5). Histopathologically, an ulcer was also identified in the wall of the transverse colon. The bottom of the ulcer was connected to the mesentery by an abscess cavity (Fig. 6a-c). There were no findings of mesenteric aneurysm and arterial sclerotic change. Abscess wall was constituted of inflamed granulation tissue with internal bleeding. The patient was diagnosed with a spontaneous mesenteric hematoma that formed a fistula to the transverse colon without any malignancy. The postoperative course was uneventful, and hence, the patient was discharged on the 10th postoperative day.

## 3 Discussion

The spontaneous mesenteric hematoma is a rare condition of unknown etiology, and it was first described in 1909 [6]. The main cause of a mesenteric hematoma is trauma [1], whereas, non-traumatic causes generally result into rupture of visceral artery aneurysms [7]. Some cases of spontaneous mesenteric hematoma were reported with potential causes, such as anticoagulation mismanagement [8,9], connective tissue disease [10], acute pancreatitis [11], and others. In about 40% of the cases, identified at the exploration could not suggest the cause of bleeding [12]. We could find 2 previous reports on a mesenteric hematoma that ruptured into the intestine [13,14]. One case presented with a fistula formed between the mesentery and the small intestine; and the other case presented with a retroperitoneal hematoma restricting the blood supply to the rectum, that resulted into ischemia and perforation. In our case, there were no histopathological findings suggestive of mesenteric aneurysm and arterial sclerotic change. The first abdominal contrast-enhanced CT indicated a mass in the mesentery with an extravasation. At the second visit, CT showed the mass containing air, and the patient had a complaint of bloody stool. Therefore, we suppose that the ischemia of the intestinal wall occurred due to pressure by the mesenteric hematoma. As a result, the fistula was formed between the mesenteric hematoma and transverse colon.

A mesenteric hematoma presents with non-specific symptoms, such as abdominal pain, vomiting, abdominal masses, diarrhea, and melena. Therefore, it is usually identified by an abdominal contrast-enhanced CT, ultrasound, or MRI [4]. The diagnosis of this disease is difficult, because it presents with similar findings to those of other diseases such as mesenteric tumor, abscess, or aneurysm. In our case, we thought that it was possible to diagnose the mesenteric hematoma because the extravasation in the mass was confirmed.

The treatment of a mesenteric hematoma depends on the clinical stability of the patients. The patients whose vital signs are unstable or those who do not respond to fluid resuscitation should be given an emergency operation. On the other hand, the patients in stable condition after the resuscitation can be treated with the non-operative methods [4]. In this case, based on many findings, such as stable vital signs, mild anemia, and improvement in the abdominal pain, we confirmed that the hemostasis was maintained. Therefore, we commenced with the conservative management. However, considering the possibility of sudden change in the patients, careful follow-up is necessary to assess fistula formation and perforation when conservative therapy is selected. Considering early hemostasis and shorter operation time, it is better to perform open laparotomy on the patients whose vital signs are unstable or those who do not respond to fluid resuscitation. On the other hand, if the patient is in stable condition, a laparoscopic approach may be useful because of many advantages, such as reduction in wound pain, in the rate of respiratory complications. In addition, we can perform the surgery safely due to a better field of view of the abdominal cavity. In this case, we chose the laparoscopic approach because the patient’s clinical condition was stable. HALS can touch the abdominal organs directly; therefore, we could proceed with the surgery while safely checking them.

We could not find any previous reports on a spontaneous mesenteric hematoma resected by a laparoscopic surgery. This is the first report on the laparoscopic resection of a spontaneous transverse mesenteric hematoma with a fistula.

## 4 Conclusions

We experienced a rare case of mesenteric hematoma with a fistula to the transverse colon. A laparoscopic surgery is useful in the cases where the patients are clinically stable.

## Conflicts of interest

None of the authors have any commercial or financial involvement in connection with this study that represents or appears to represent any conflicts of interest.

## Funding

This research received no specific grant from any funding agency in the public, commercial, or not-for-profit sectors.

## Ethical approval

Ethical approval from our institution OJH-201854.

## Consent

Written informed consent was obtained from the patient for publication of this case report and any accompanying images.

## Author contribution

All authors in this manuscript contributed to the interpretation of data, and drafting and writing of this manuscript. Tomoaki Bekki is first author of this paper. Takuya Yano is corresponding author of this paper. Tomoaki Bekki, Takuya Yano and Masahiro Nakahara conceived and designed the study and drafted the manuscript. Tomoaki Bekki first diagnosed. Tomoaki Bekki, Takuya Yano and Masahiro Nakahara were engaged in patient’s care in our hospital including surgery.

Hiroyuki Egi contributed to study concept, and review of the final manuscript and submission of the paper. All the authors read and approved the final manuscript.

## Registration of research studies

The manuscript does not report the result of an experimental investigation or research on human subjects.

## Guarantor

Takuya Yano.

## Provenance and peer review

Not commissioned, externally peer-reviewed.
